# Masking the Pathogen: Evolutionary Strategies of Fungi and Their Bacterial Counterparts

**DOI:** 10.3390/jof1030397

**Published:** 2015-12-10

**Authors:** Yoon-Dong Park, Peter R. Williamson

**Affiliations:** Laboratory of Clinical Infectious Diseases, National Institute of Allergy and Infectious Diseases, National Institutes of Health, 9000 Rockville Pike, Building 10, Rm 11N222, MSC 1888, Bethesda, MD 20892, USA; E-Mail: parky4@mail.nih.gov

**Keywords:** capsule, fungal immunity, *Cryptococcus*, *Histoplasma*, *Blastomyces*

## Abstract

Pathogens reduce immune recognition of their cell surfaces using a variety of inert structural polysaccharides. For example, capsular polysaccharides play critical roles in microbial survival strategies. Capsules are widely distributed among bacterial species, but relatively rare in eukaryotic microorganisms, where they have evolved considerable complexity in structure and regulation and are exemplified by that of the HIV/AIDS-related fungus *Cryptococcus neoformans*. Endemic fungi that affect normal hosts such as *Histoplasma capsulatum* and *Blastomyces dermatitidis* have also evolved protective polysaccharide coverings in the form of immunologically inert α-(1,3)-glucan polysaccharides to protect their more immunogenic β-(1,3)-glucan-containing cell walls. In this review we provide a comparative update on bacterial and fungal capsular structures and immunogenic properties as well as the polysaccharide masking strategies of endemic fungal pathogens.

## 1. Introduction

Surface structures delineating the host-pathogen interface are critical to the outcome of microbial infections. The external structures of microbes include viral envelopes, parasite surface protectants, as well as capsules and cell walls of bacteria and fungi. These protein, lipid, or carbohydrate masks range from relatively simple oligomers to complex macromolecules or extensive polymers [1]. Polysaccharide capsules (PC) are important virulence factors in many pathogenic microbes that provide a protective coat against host immunity. They are highly diverse hydrated structures that provide microbes with a key defense against the host immune system [2]. For example, bacterial capsules confer resistance to complement-mediated opsonophagocytosis [3] and are an important property of highly virulent bacteria such as *Streptococcus pneumoniae* and *Neisseria meningitidis* [4]. Among fungal pathogens, the opportunistic pathogen *Cryptococcus neoformans* is unique in having a large PC with potent anti-phagocytic properties [5]. *C. neoformans* is a major opportunistic infection, each year causing approximately a half a million cases of meningitis in HIV/AIDS patients globally, and whose PC is a major virulence factor. Despite its importance in pathogenesis, many aspects of capsular architecture in both bacteria and fungus remain incompletely understood. Other fungal pathogens including the endemic fungi, *Histoplasma capsulatum* and *Blastomyces dermatitidis* which cause pulmonary disease in immunocompetent hosts, have non-capsular masking strategies that incorporate immunotolerant carbohydrates within their cell wall. In this review we will provide an overview of protective capsule structures and related masking mechanisms comparing the fungal pathogen, *Cryptococcus neoformans* with representative bacterial and fungal pathogens.

## 2. Sugar-Coated Killers: Capsular Structures of Bacteria and a Pathogenic Fungus

In prokaryotes, the cell capsule is composed of an extensive polysaccharide (PS) layer that lies outside the cell envelope or cell wall, attached to the cell periphery via covalent attachments to either phospholipid or lipid-A molecules. Bacterial capsules are distinct from the second lipid membrane or bacterial outer membrane, which contains lipopolysaccharides and lipoproteins. In addition, an amorphous viscid secretion may diffuse from the capsular matrix into the surrounding medium and remains as a loose un-demarcated slime layer, constituting a water-rich gel which protects the bacteria against desiccation, and excludes other bacteria as well as viruses and hydrophobic toxic materials such as detergents [6]. The extracellular structure can be visualized using India ink, whose microparticles are excluded due to the extensive PS layer surrounding the cell, resulting in a clear zone surrounding the cell wall [7,8]. Bacterial capsules are made up of long PS chains, which are typically negatively-charged and generate a highly hydrated capsular layer. When examined under the microscope, capsules appear swollen due to an increase in refractive index and this is the basis of the Quellung reaction [8]. Some bacterial capsules too small to be seen with an ordinary microscope, such as the M protein of *Streptococcus pyogenes*, are referred to as microcapsules [9].

**Figure 1:**
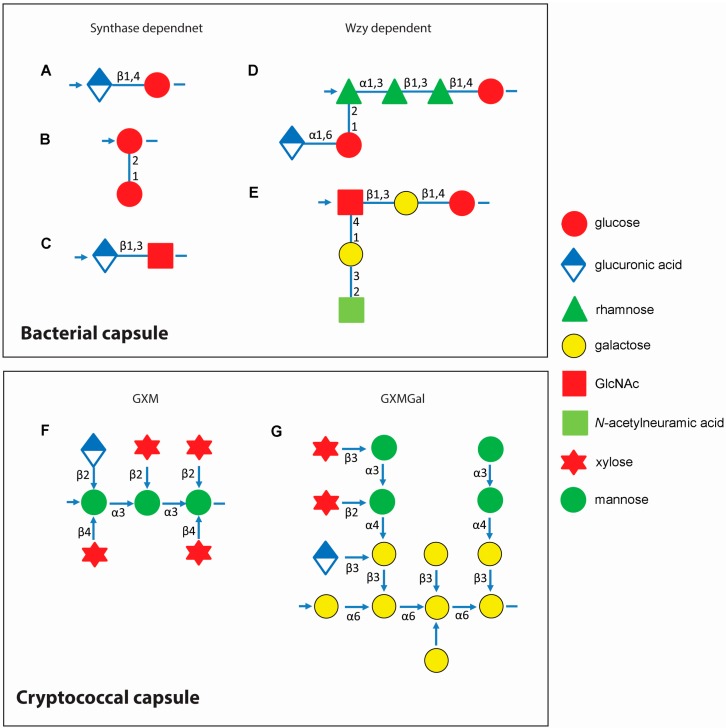
Capsule repeat units of *Streptococcus* spp. and *Cryptococcus neoformans*. *Upper panel*: capsule repeat units of *Streptococcus pneumoniae* serotype 3 (**A**); *S. pneumoniae* serotype 37 (**B**); *S. pyogenes* HA (**C**); *S. pneumoniae* serotype 2 (**D**); and *S. agalactiae* serotype III (**E**); Structures for additional *S. pneumoniae* capsules can be found in Bently *et al.* [18] and van Dam *et al*. [19]. This figure is adapted from Yother [17]. *Lower panel*: capsule repeat units of *C. neoformans*. Left, one of the six structural reporter groups that are found in varying proportions in glucuronoxylomannan (GXM) within strains of *C. neoformans*, defined by Cherniak *et al.* [20] Linkages between sugars are printed next to the arrows connecting monosaccharides (**F**); Right, the structure of glucuronoxylomannogalactan (GXMGal) (**G**). Lower panel is adapted from Doering [1]. Arrows indicate direction of polysaccharide synthesis.

Bacterial capsules are widely distributed and are found in Gram-negative bacteria, including strains of *Escherichia coli* [10], *Klebsiella pneumoniae* [11], *Haemophilus influenzae* [12], and *Pseudomonas aeruginosa* [13]. Some Gram-positive bacteria also express capsule: *Bacillus megaterium*, synthesizes a capsule composed of polypeptides and PSs [14]. *Streptococcus pyogenes* also synthesizes a hyaluronic acid capsule, and *Streptococcus pneumoniae* and *Streptococcus agalactiae* produces nine antigenic types of PC that contain sialic acid (Ia, Ib, II, III, IV, V, VI, VII, and VIII) [15]. These extracellular shields can be quite extensive and negatively charged surfaces may improve hydration and pathogen dispersion. For example, in some *E. coli* strains, capsule layers can extend from the cell surface for approximately 100–400 nm, and are formed by glycan chains more than 200 sugars long [7,16]. Bacterial capsules are formed primarily from long-chain PSs with repeat-unit structures. Among the two archetypes of primary biosynthetic structures in *S. pneumoniae*, synthase-dependent polymers are relatively simple structures, with only one or two repeating sugars. A second, Wzy-dependent structure occurs widely in both gram-positive and gram-negative bacteria and is composed of multiple different sugars derived from both glucose and an unusual sugar, rhamnose decorated with branching glycosidic linkages (Figure 1, upper panel). Rhamnose is distinctive from other monosaccharides such as glucose in that it is a naturally occurring deoxy sugar, and occurs in its natural form in the l-form, where most naturally occurring sugars such as glucose are in the d-form (see the excellent review by Yother [17]).

As hydrated structures, capsules assist in evasion of the host immune response [21] and could theoretically protect bacterial strains from desiccation. However, in pathogenic bacteria such as *Streptococcus pneumoniae*, desiccation tolerance in the *ex vivo* environment is not dependent on the PC [22]. This suggests the capsule of some bacteria is not an important environmental protective factor and more likely evolved under the selective pressure from host defenses. This also suggests that the simplicity of the capsule structure of some bacteria may not allow significant binding of water during environmental drying. In contrast, environmental desiccation resistance appears to be a more important function of the larger and more complex fungal capsule [23,24]. For example, Aksenov and co-workers demonstrated that the fungal capsule delays desiccation and speeds water uptake by comparison of wild-type and hypocapsulated strains of *Cryptococcus diffluens* using spin-echo nuclear magnetic resonance [24]. Yeast capsules are also thought to facilitate dispersal and nutrient access in the environment [25]. This suggests that the complexity of fungal capsules such as that of *Cryptococcus* may allow significant binding of water and protection during environmental drying.

The fungal capsule of *Cryptococcus neoformans* is also not visible by regular microscopy, but similar to bacterial capsules, can be viewed by India ink microscopy as an extensive clear zone surrounding the cell wall. It can also be robustly observed by other microscopic techniques, such as scanning electron microscopy and tagged fluorescent molecules [26]. The *C. neoformans* capsule possesses some functional similarities to those of encapsulated bacteria such as *Streptococcus pneumoniae* and *Haemophilus influenzae* [27,28], and is known to share antigenic determinants with certain pneumococcal PSs [29,30]. In addition, the presence of capsule has been implicated in biofilm formation, which correlates with the ability of capsular PS to bind polystyrene solid supports [31]. The primary structure of the cryptococcal capsule has been well characterized [32] and summarized in review [1,26,33]. The capsule is a hydrated PS gel, constituted by large PS polymers including glucuronoxylomannan (GXM) which represents almost 90% of the total capsule. Unlike bacterial capsules that are formed primarily from glucose-derived subunits, the cryptococcal backbone consists of mannose residues that are α-1,3 linked and contain xylosyl and glucuronyl side groups (Figure 1, lower panel). Roughly two of every three mannose residues are also 6-*O*-acetylated [34,35,36], with a predominance of unbranched mannose but with some acetylation, substituted with glucuronic acid [37]. Similar to bacterial capsules, the cryptococcal capsule confers a strong negative charge that helps in capsule hydration by virtue of glucuronic acid residues decorating its main PS backbone [38]. The glucuronoxylomannanogalactan (GXMGal) constitutes about 7% of the capsular mass and has a more elaborate structure than GXM [39]. It is built on an α-1,6-linked galactose backbone containing side chains of different lengths on alternate galactose residues [32] (Figure 1, lower panel). In addition, a small proportion of mannoproteins (MPs) have been identified. MPs are heavily mannosylated partially secreted proteins. Most have unknown functions although one of the products, the Cig1 mannoprotein, possesses iron acquisition functions at the cell surface. Iron acquisition has been shown to be important in virulence and undergoes micro-evolutionary changes during the conversion of environment strains to pathogenic ones [40]. GXM, GXMGal and MPs are also released into the extracellular milieu; however, MPs are not covalently linked to GXM and GalXM. Mannoproteins play an important role in immune recognition of the fungus, playing a role in stimulation of dendritic cells [41] and optimizing T-cell reactivity [41].

## 3. Assembly of Capsular Components Show Evolution in Complexity

### 3.1. Bacterial Capsule Synthesis

Over the past two decades, genetic and biochemical analyses of polysaccharide (PS), as well as capsules and exopolysaccharides from gram-positive and gram-negative bacteria have demonstrated that many details of polymer synthesis and regulation are broadly shared. Central to the understanding of many of these processes has been the elucidation of lipopolysaccharide *O*-antigen synthesis in Gram-negative bacteria and the identification of three primary biosynthetic pathways: Wzy-, synthase-, and ABC transporter dependent [42,43]. The Wzy and synthase mechanisms are named for the polymerases of the respective pathways (Figure 1, lower panel); the ABC transporter pathway is named for mechanisms involved in exporting the PS to the cell surface [17]. The Wzy-, synthase-, and ABC transporter-dependent mechanisms occur in Gram-negative bacteria, whereas the Wzy- and synthase-dependent mechanisms are present only in gram-positive bacteria [17]. Capsular synthesis is similar, utilizing enzymes which are usually firmly associated with the cell via covalent linkages, and exopolysaccharides, which are released or only loosely associated with the cell. The more complex capsules synthesized by the Wzy polymerase are assembled on the inner face of the cytoplasmic membrane and then a Wzy flippase transports the PS to the membrane outer face where final assembly is dependent on extracellular Wzy polymerase activity.

In ABC transporter-dependent assembly, capsular PS is synthesized in the cytoplasm and exported by a specific ABC transporter, composed of two identical nucleotide-binding domain polypeptides and two integral membrane polypeptides [44]. Completion of transport from the periplasm to the cell surface requires two other defining components: a PS co-polymerase family member and an outer membrane PS protein [45]. The repeat-unit PS structure is assembled through the action of glycosyltransferase enzyme activities. The glycosyltransferases catalyze the transfer of a sugar from an activated donor to a substrate, which can be protein, lipid, or another carbohydrate [46]. In the ABC transporter-dependent pathway, specific sugars are transferred to the non-reducing end of a growing PS glycan [47,48,49]. In *Streptococcus pneumoniae*, the serotype 3 capsule is cell associated via linkage to phosphatidylglycerol or interactions with the synthase but not by linkage to the peptidoglycan. Chain length is regulated by the ratio of uridine diphosphate glucose (UDP-Glc) to uridine 5’-diphosphogluucuronic acid (UDP-GlcUA) and, ultimately, the UDP-GlcUA concentration, producing a tremendous variety of PS structures present in bacterial capsules [21,50].

Bacterial strain serogroups are determined by the structural variability of surface PSs. For example, *Escherichia coli* strains and *Klebsiella* species are divided into 160 and 72 serogroups, respectively, based on differing surface PSs [10,51]. In addition, strains of *Streptococcus pneumoniae* produce 90 different capsular PSs, distinguished by using specific antiserum that recognizes chemical differences in the capsules [18]. Multiple cross-absorptions select for sets of serogroup-specific anti-serum useful as diagnostic and typing tools, allowing the categorization of strains having immunochemical differences between the various pneumococcal capsular PSs. Comparisons of polysaccharide capsule (PC) loci suggest a variety of genetic mechanisms and show that the core machinery involved in the synthesis and polymerization of repeat unit vary widely and are often non-homologous between serotypes. However, the evolutionary timescales and genetic events leading to the emergence of novel serogroups remain poorly understood. Nevertheless, the extensive genetic diversity suggests strong evolutionary selective pressures brought out by the host immune response. Under such pressures, new serotypes have been generated by diverse mechanisms including the introduction of new PC genes into pneumococci by lateral gene transfer from other species [18].

### 3.2. Cryptococcal Capsule Synthesis

Much like their bacterial counterparts, strain identification of cryptococcal subgroups has historically relied on specific antisera prepared in rabbits [52]. The cryptococcal serum and CSF antigen latex agglutination (LA) tests have been instrumental in the clinical diagnosis of fungal infections caused by cryptococcal strains, having high sensitivity and specificity [53], although recently the LA has been replaced by an even more sensitive lateral flow assay using anti-capsular monoclonal antibodies [54,55].

In addition to its use in clinical detection, the extracellular capsules of *C. neoformans* are also crucial for success of the pathogen [1]. Thus, understanding capsular synthesis may help identify fungal targets for new therapies which do not require uptake of inhibitory materials into the interior of the fungus. Extensive work by numerous investigators has provided key insights into synthesis of the capsular primary structure [32]. Nuclear magnetic resonance spectroscopy (NMR) analysis of shed glucuronoxylomannan (GXM) has defined six structural reporter groups, based on a composition of xylose, mannose, and galactose, which occur in reproducible combinations in various strains [39]. The repeating structures of GXM and GXMGal suggest that individual subunits are synthesized and linked together intracellularly, reminiscent of bacterial peptidoglycan synthesis [1].

Many cryptococcal genes involved in capsule synthesis have been identified. Some of the first genes involved in capsule biosynthesis were identified sequentially by Chang, Kwon-Chung and co-workers: *CAP59* [56], *CAP64* [57], *CAP60* [58], and *CAP10* [59]. Deletion of these *CAP* genes by homologous recombination result in acapsular cells that are predominantly avirulent in mice and complementation restored capsule expression and virulence. These genes are specific to *Cryptococcus* and suggest unique fungal processes involved in capsular synthesis. For example, while bacterial capsular PSs are synthesized by plasma membrane glycosyltransferases and assembled extracellularly, the large component GXM of the *C. neoformans* capsule requires cellular transport as it is synthesized intracellularly within the Golgi apparatus [60,61,62]. For this process, GXM trafficking to the cell surface requires transport of assembled structures within PS-containing vesicles [60,61,63] which cross the plasma membrane and cell wall in a *SEC6*-dependent manner [64], releasing their content into the extracellular space.

Unlike the heavy reliance on glucose-derived subunits in bacterial capsules, mannose is the most abundant sugar unit in cryptococcal GXM. The *C. neoformans* gene, named *MAN1*, encoding for phosphomannose isomerase was characterized by Perfect and co-workers [65]. The *man1*∆ mutant had a poor ability to form capsule, produced reduced levels of exopolysaccharide, and exhibited attenuated virulence in mammalian models of cryptococcosis [65]. Glucuronic acid is the second sugar unit of GXM and is contained in PS branches rather than within the chain itself. The gene encoding the enzyme, *UGD1*, was characterized by Doering and co-workers. The cryptococcal enzyme is a dimer whose activity is regulated by NAD^+^- and UDP-glucose binding [66]. The *ugd1*∆ mutant is acapsular with alterations in cell integrity, morphological defects at the bud neck, lack of growth in an animal model and enhanced sensitivity to temperature, detergent, NaCl, and sorbitol [67,68]. Xylose is the third monosaccharide component of GXM and is utilized by fungal glycosyltransferases in the form of UDP-xylose, which is synthesized from the decarboxylation of UDP-glucuronic acid by UDP-glucuronic acid decarboxylase [69]. GXM *O*-acetylation provides capsular branching for the assembled structure, dependent on the *O*-acetyltransferase enzyme Cas1 [37]. Assembly of these branched units then forms an extensively branched, hydrated gel that surrounds each fungal cell.

Despite its complexity and size, the cryptococcal capsule architecture is dynamic under differing physiological conditions. For example, capsule enlargement occurs in a variety of capsule-inducing media which modify nutrients and environmental stressors [70] and a dramatic enlargement is observed during mammalian infections [70,71]. Numerous regulatory proteins modulate capsule size in *C. neoformans* in response to these nutrient changes or stress conditions [56,58,59]. For example, Gpa1 of the nutrient-signaling cAMP pathway transcriptionally regulates at least nine genes for capsule synthesis or assembly, including *CAP10*, *CAP59*, and *CAP64* and the *O*-acetyltransferase and UDP-xylose synthase enzymes described above [72]. Disruption of the G-protein *CRG2* also increases cAMP levels and results in larger capsules, suggesting that Crg2 negatively regulates the Gpa1-cAMP pathway [73], involved in capsular regulation. Many of the conserved components of the cAMP-signaling cascade play a role in capsule regulation and have been characterized including the Gα protein (Gpa1), G protein-coupled receptor (Gpr4), adenylyl cyclase (Cac1), and the protein kinase A catalytic subunit (Pka1) [74,75,76]. Changes in distinct structural regions are also evident during capsule induction, suggested by differential extraction of capsular layers by the solvent, dimethyl sulfoxide (DMSO). DMSO extraction of intact cells with large, mature capsules releases an outer layer of capsular particles. A second, internal DMSO-resistant region remains cell associated [14,77]. Recent data using a number of elegant and detailed biophysical methods has suggested that the outer DMSO-extractable layer is assembled by non-covalent binding of PS fibrils that have a branched structure [78]. This mechanism of capsule assembly is akin to building an immunologically protective “igloo” from assembled blocks and is unique to fungal capsular structure. Current models suggest that the primary structure is synthesized intracellularly [1] and that secreted proteins may be involved in formation of capsular tertiary structure.

**Figure 2:**
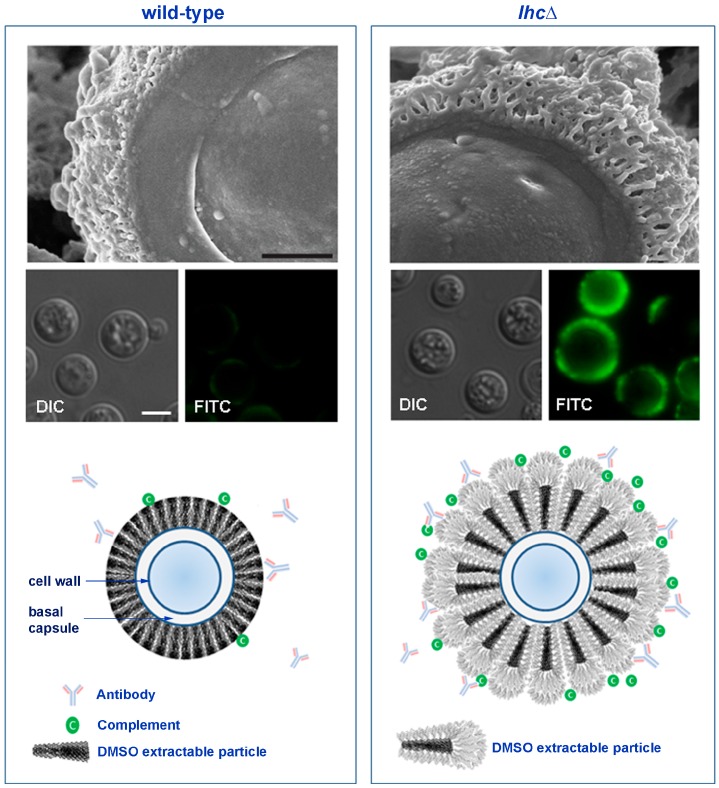
Wild-type and *lhc1*Δ mutant strains of *C. neoformans* differ in capsule and antibody binding. *Upper panel*: Three dimensional ultrastructure of *C. neoformans* capsule. *C. neoformans* wild-type (H99) and lactonohydrolase knock out (*lhc1*Δ) cells were subjected to Cryo-Scanning Electron Microscopy (Cryo-SEM). Bar = 500 nm. *Middle panel*: Antibodies to C3 complement demonstrate reduced binding of a key complement component in WT cells. Indicated strains were prepared and stained with anti-C3 complement monoclonal antibodies. Bar = 5 µm. *Lower panel*: Scheme of working model of capsule modification by Lhc1 (adapted from Park *et al*. [79]). In the absence of Lhc1, unprocessed PS units with increased branching provide antigenic sites for antibody and complement binding, resulting in increased phagocytosis and reduced virulence. Lhc1 expression results in a compact capsule less hydrated surface, resulting in better exclusion of opsonizing antibodies and complement, reducing phagocytosis and increasing virulence.

One of these modulating extracellular proteins has recently been described which provides molecular insights into this unique fungal process. Using a targeted proteomics approach, a capsular lactonohydrolase (Lhc1) of *C. neoformans* was identified as an extracellular component of cryptococcal capsule and characterized by using a targeted mutant strain. The mutant strain demonstrated a larger capsule, more permeable to fluorescent dextran particles. In addition, the mutant isolated PS was larger, more hydrated and branched, evidenced by an altered biophysical properties including capsule nuclear magnetic spectra, zeta potential and PS hydrodynamic dimensions [79]. Fluorescence tagging of Lhc1 also suggested production of the enzyme during capsule induction at the outer region of the capsule thought to represent the inducible outer layer [80]. The *lhc1*∆ mutant also demonstrated increased complement and antibody-dependent phagocytosis by the macrophage cell line J774.16 cells and reduced virulence in mice that could be reversed by depletion of complement using cobra-venom or by reconstitution of the *LHC1* gene (Figure 2, middle panel) [79]. The location of a hydrolytic lactonohydrolase within the PS capsular structure and the larger size of the PS particles in the mutant strain suggest that the enzyme either directly or indirectly plays a role in remodeling secreted PS fibrils. Hydrolysis of outer branching units within this PS surface structure by Lhc1 in wild-type cells reduced the size of the capsular PS compared to that of the *lhc1*Δ mutant, with reductions in overall branching (Figure 2, left lower panel). This resulted in reduced hydration of the capsular structure with a smaller radius of hydration and less negative zeta potential in the wild-type cells, resulting in a tightly compacted capsule. These structural changes were supported by cryo-scanning electron microscopy which demonstrated truncated fibrils in WT cells, whereas the mutant strain demonstrated a much more open lattice composed of larger fibrils, representing the larger PS demonstrated by the polydispersity measurements (Figure 2, right upper panel). Lhc1 expression thus results in a compacted structure that reduces binding of important opsonizing components of the mammalian immune system (Figure 2, left middle panel) and serves to increase the virulence of the fungus. Such extracellular modifications have the potential to potentiate strain-to-strain variability as well as to rapidly modulate capsular architecture and the host immune response during mammalian infection.

### 3.3. Non-Pathogenic Fungal Capsules are Less Protective

Among the genus *Cryptococcus*, the species mainly responsible for disease in human and animals are *Cryptococcus neoformans* and *Cryptococcus gattii* [81]. However, in recent years there has been an increased incidence of infections caused by related species, including *Cryptococcus laurentii* and *Cryptococcus albidus*, responsible for a predominance of non-*neoformans*, non-*gattii* cases [82,83,84,85]. Otherwise, *Tremella mesenterica*, a close phylogenetic relative to genus *Cryptococcus*, possesses a similar capsule but does not cause disease [86]. Several other fungi include *Malassezia furfur* [87], *Rhinosporidium seeberi* [88], *Trichosporon beigelii* [89], *Blastocystis hominis* [90], and *Sporothrix schenckii* [91], have capsule-like structures, but have not been well characterized. Understanding structural elements that differentiate pathogenic from non-pathogenic fungal species may thus be important for ascertaining the virulence potential of various members of this genus. Recently, *Cryptococcus liquefaciens* was recovered from the giant snail *Achatina fulica* by the Frases group [92]. *C. liquefaciens* is a member of the genus *Cryptococcus*, but there are no reported cases of human disease by this species. Frases and co-workers demonstrated that *C. liquefaciens* produces a similar PS capsule to that of pathogenic members of the genus *Cryptococcus* in many aspects, but the capsular and exo-PS have structural differences that leave them less efficient in protecting the yeast. Isolated capsular and exo-PS from *C. liquefaciens* contained a similar monosaccharide composition when determined by GS-MS, but the ratios of monosaccharides differed between the two species. Glucose was also detected, which may reflect the co-extraction of glucans, which are frequently found in DMSO extracts of *C. neoformans* [93], and the amount of glucose was higher in *C. liquefaciens* PS fractions. *N*-acetylglucosamine was detectable by GS-MS and labeled by fluorescent lectin, indicating its presence in the *C. liquefaciens* capsule. *C. liquefaciens* also has a capsule visible in India ink preparations that is efficiently labeled by three antibodies generated to specific *C. neoformans* capsular antigens. Chemical analysis also demonstrated that the *C. liquefaciens* capsule contains mannose, xylose, glucose, glucuronic acid, galactose and *N*-acetylglucosamine. In addition, physical and chemical analysis of the *C. liquefaciens* PSs revealed significant differences in viscosity, elastic properties and macromolecular structure from that of *C. neoformans* [92]. Both *C. neoformans* and *C. liquefaciens* were phagocytosed at similar rates, but the intracellular survival of *C. neoformans* was significantly greater than that of *C. liquefaciens* [92]. This suggests that morphologically similar capsules by light microscopy may have major differences in physical and protective properties, but the *C. liquefaciens* PS is not sufficient to protect the fungus from predation. Furthermore, the ability of specific surface PS elements to block interactions with host phagocytes may be a key determinant for the ability of fungal cells to cause disease in mammalian hosts and differentiates opportunistic pathogens from exclusively environmental microbes. In summary, the capsular structures in pathogenic *Cryptococcus* species and exclusively environmental species share similar features, but also manifest significant difference that could influence their potential for virulence.

## 4. Function Follows Form: Capsule Alters Host Immune Responses

### 4.1. Bacterial Capsules and Host Defense

A primary function of capsules in pathogenic bacteria is to shield the bacterial surface from interactions with components of the host immune system and prevent either opsonophagocytosis or, in Gram-negative bacteria, complement-mediated lysis [17]. Immunity to one capsule type does not result in immunity to the other types due to the unique structures of the PC described above. The bacterial capsule is also considered a virulence factor as it enhances the ability of bacteria to cause disease by preventing phagocytosis. In addition, in neutropenic patients capsule can also protect bacteria from engulfment by host macrophages [94]. In many bacteria, therefore, the capsule is required for evasion of the host immune system; indeed, acapsular strains are generally non-pathogenic. Not surprisingly for the survival of the bacteria, capsular material is poorly antigenic but can be made suitable for a vaccine response by conjugation with T-cell augmenting proteins such as tetanus toxoid [95,96,97,98]. Vaccination using capsular material has thus become effective against a number of highly pathogenic organisms including *Haemophilus influenzae* type b, *Streptococcus pneumoniae*, and *Neisseria meningititidis* [99], resulting in considerable public health impact against these diseases [46,100,101].

### 4.2. Innate Shielding of Fungi

Protection of the immunocompetent host from systemic infections by unencapsulated pathogenic fungi such as *Candida albicans* and *Aspergillus fumigatus* is facilitated by the innate immune system, initiated after binding of pattern recognition receptors including the C-type lectin receptors, Dectin-1 [102], pentraxin-3 [103], Toll-like receptors (principally TLR2/6, TLR4 and TLR9) and nucleotide-binding oligomerization domain (NOD)-like receptors [104,105]. Exposed fungal cell wall constituents such as β-glucans are readily recognized, resulting in inflammation and control of these fungal infections in normal human hosts. Formation of biofilms by organisms such as *C. albicans* results in reduced recognition by pattern recognition receptors [106] but still require an altered host for successful infection. Thus, susceptibility to these fungal diseases have been linked to host defects brought on principally by iatrogenic neutropenia from immunosuppression as a result of cancer chemotherapy, transplant conditioning or anti-inflammatory treatment of auto-immune diseases [107]. In addition, polymorphisms within immune receptors have been identified as secondary contributors such as Dectin-1 Y238X with aspergillosis in transplant patients [108] and TLR1 polymorphisms in candidemia [109] as well as an association of aspergillosis in transplants [110] with defects in signaling pathways related to these receptors including Dectin-1-related CARD9 [111]. Phagocytosis by macrophages and neutrophils is facilitated by deposition of complement, C3 and C5 followed by binding to complement receptors, deficiency of which results in severe disease in murine models [112]. More severe genetic defects in lectin receptors, such as CARD9 deficiency, result in rare causes of these fungal infections without immunosuppressant therapy [111,113].

**Figure 3:**
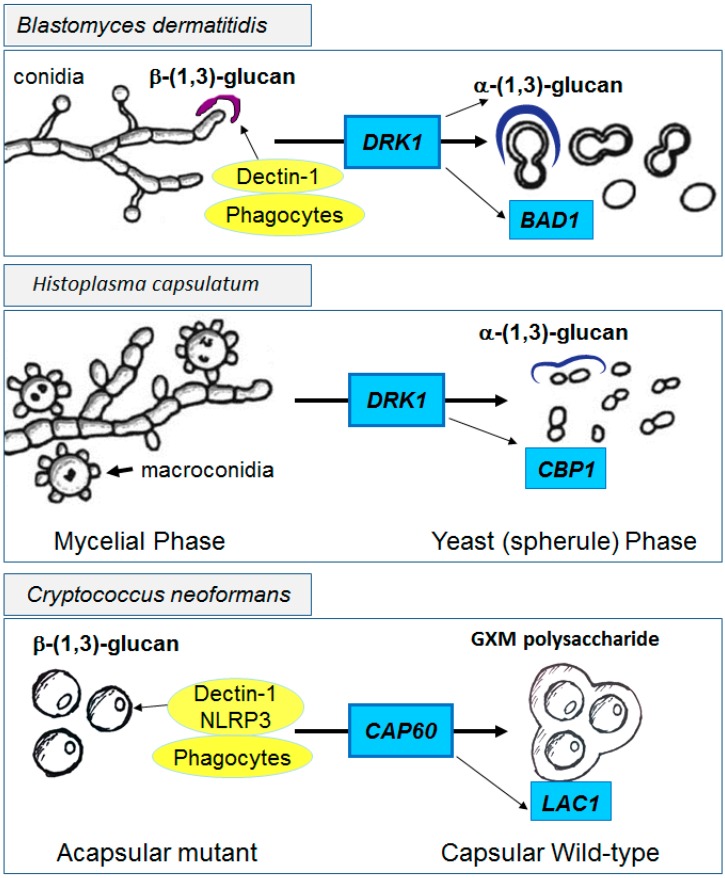
Functional effects of immune shielding by pathogenic fungi. Unshielded fungal forms such as the mycelial phases of *B. dermatitidis*, *H. capsulatum* or acapsular mutants of *C. neoformans* (left panels) provide exposed cell wall β-(1,3)-glucans for innate immune recognition. In contrast, after induction of the yeast forms of dimorphs by *DRK1* or with expression of capsular genes such as *CAP60* in *C. neoformans*, shielded yeast forms display reduced immune recognition and increased virulence (right panels). Additional virulence genes such as *BAD1*, *CBP1* and *LAC1* contribute to pathogenicity in the three fungi, respectively.

In contrast to fungi that infect immune suppressed hosts, fungi that infect normal hosts such as *Blastomyces* and *Histoplasma* have evolved other highly effective shielding mechanisms such as cell wall α-(1,3)-glucans which are accompanied by reduced cell wall β-(1,3)-glucans and are induced during a switch from non-pathogenic environmental hyphal forms to pathogenic yeast forms, regulated by the histidine kinase Drk1 [114] (Figure 3 Top and middle panels). Deposition of α-(1,3)-glucans within the outermost layer of the cell wall result in reduced innate recognition, facilitating successful infections even in hosts with intact immunity [115]. Additional proteins such as the adhesin and anti-phagocytic protein Bad1 in *B. dermatitidis* or the calcium binding protein *CBP1* in *Histoplasma capsulatum* further facilitate virulence in the intact host [116,117]. In contrast, hyphal forms that exist at lower temperatures in the environment do not highly express α-glucans in their cell walls, making these forms much less virulent.

### 4.3. The Cryptococcal Immune Shield

In the same way, unencapsulated cryptococcal cells are easily recognized via innate receptors that bind cell wall β-glucans [118], resulting in NLRP3 inflammasome activation [119], rapid phagocytosis [120] and clearance; thus, acapsular mutants of *Cryptococcus* are rarely isolated from infected patients (Figure 3 lower panel). However, shielding of β-glucans by the PC, facilitated by trafficking proteins such as Cap60 which bring components to the outer cell wall for assembly [61], results in reduced recognition and phagocytosis by immune cells [121,122] and the complement system [123]. Reduction of complement binding and opsonization is facilitated by capsular remodeling by proteins such as Lhc1 described above, minimizing the deposition of antibodies and complement [79]. The antiphagocytic properties of the PC are also potentiated by other cryptococcal factors such as the anti-phagocytic protein App1 which blocks complement 2 and 3 receptors (CR2 and CR3) [124] and virulence potentiated by the immune modulating cell wall and secreted laccase [125]. However, *Cryptococcus* is not primarily a pathogen of normal hosts; thus, recognition by Toll-like receptors [126,127], mannose receptors [41], β-glucan receptors [118,128] and complement [121,129,130] are sufficient to mediate clearance of even encapsulated strains in most immunocompetent hosts in the presence of intact T-cell and macrophage signaling. Interestingly, the relative shielding from the innate immune system by the capsule relative to adaptive immunity results in a unique immune susceptibility of *Cryptococcus* to adaptive defects. Thus, immunodeficiencies resulting in disseminated infections with *Candida* or *Aspergillus* do not strongly overlap with those resulting in cryptococcosis and vice versa.

Defects in T-cell immunity are a strong risk factor for cryptococcal infections [131]. Fungal GXM partially inhibits leukocyte migration of many types of cells, including T-cells [132], synergizing with acquired defects in T-cell immunity by infections with HIV, treatment with chemotherapy or steroids [133,134,135] or in the presence of idiopathic CD4 lymphopenia [136]. Secreted GXM also binds the inhibitory receptor FcγIIB [137] where it leads to induction of the death receptor FasL, activating apoptosis in activated T-cells via the FasL/Fas pathway through upregulation by JNK and p38 activation [138,139,140]. In addition, GXM induces macrophage apoptosis and inhibitory macrophage cytokines such as IL-10 [141,142]. This fungal-derived macrophage defect thus is able to synergize with acquired defects in macrophage activation such as in patients with autoantibodies to the macrophage-inducing granulocyte-monocyte colony stimulating factor (anti-GMCSF), resulting in brain infections by *C. neoformans* and *C. gattii* in these susceptible patients [143,144]. However, while Lhc1-dependent capsular modifications lead to partial inhibition of complement and antibody deposition, these effects do not appear to synergize strongly with human complement or antibody deficiencies [145], although an ancillary role in HIV for both peripheral blood IgM memory B-cell levels and GXM-binding IgM were found to play a role in susceptibility to cryptococcosis [146]. Synergizing with the shielding effects of the capsule, the PS component GXM can be released into the environment where it induces potent shedding of L-selectin from the surface of neutrophils [147], limiting chemotaxis and adhesion to endothelial cells [148,149], perhaps explaining the paucity of neutrophil recruitment and participation in human cryptococcal infections. Indeed, while genetic defects in monocyte-macrophage signaling such as GATA2 have been associated with cryptococcal infections [150], neutropenia is not a strong risk factor for infections with this organism.

## 5. Evolutionary Pressures in the Host and Environment: The Red Queen Paradigm

Bacterial virulence of capsular organisms such as *S. pneumoniae* is highly determined by evolutionary pressure within the host environment as the organism is a frequent mucosal colonizer and undergoes person-to-person spread [151]. The “Red Queen Paradigm” has been used as a description of this phenomenon, taken from Carroll’s novel, “Alice in Wonderland” where the Red Queen, grabbing Alice as she emerged from the looking glass, remarked, “it would take all the running (she) could do, to keep in the same place.” [152,153]. The paradigm suggests an evolutionary struggle that occurs as a pathogen tries to surmount host immunity, sometimes succeeding against individuals but not the entire population, resulting in successful survival of both populations within the host-pathogen niche. Perturbations in host responses, such as antibody repertoire can thus result in evolutionary-driven changes in targeted bacterial antigens [154]. This can occur through vertical evolutionary changes passed in subsequent generations of exposed pathogens, or though horizontal deletion of susceptible strains [155]. The former is more common after antibiotic exposure such as the emergence and dissemination of the internationally prevalent β-lactamase resistant PMEN2 strain [156]. Horizontal deletion of strains has been prominent after changes in population immunity such as the emergence of the serotype 19A capsule “escape” strain after widespread adoption of conjugate pneumococcal strains that did not contain the 19A capsular antigen [157]. However, the two have potential synergy, as the recent emergence of highly antibiotic resistant carbapenimase-producing gram negative bacteria have been hastened by the merging of an antibiotic resistance plasmid within a *Klebsiella pneumoniae* strain expressing a mucoid capsule, rendering it both hypervirulent and highly resistant [158].

The predominant ecological niche exerting selective pressure on fungi such as *Cryptococcus* is less clear because this opportunistic pathogen occupies a multitude of environments including soil [159], plants [160], the GI tract of pigeons and their excreta [161] well as the lungs and brains of mammals such as dolphins [162,163] and humans [164]. Many of these environments leave virulence “legacies” that have been adapted to cause disease in humans. For example, the CP that provides protection against enviromental desiccation, discussed above [24] may also offer protection within the soil against free living amoeba which resemble many of the features of mammalian macrophages [165]. Evolutionary pressure by free-living amoeba has also been implicated in increased virulence mechanisms of endemic fungi such as *Blastomyces* and *Histoplasma* and could have contributed to the evolution of α-glucan cell wall masking strategies in these fungi which also do not depend on human residence in their life cycles [166]. Selective pressures by predators such as amoeba may synergize with pressures of other environmental niches such as live plant seedlings where anti-fungal diphenolic compounds such as quercetin and transcinnamic acid are detoxified by a cell wall and secreted laccase enzyme [160]. Fungal laccase also facilitates robust immunomodulatory properties of the fungus in mammals including the expression of Th2 biasing prostaglandins E2 [167]; thus, a ‘dual use’ function against plant innate immunity may explain the fungi’s close environmental link to plants. Indeed, a predominant feature of this fungus may be precisely its ability to alter cellular programs to adapt to differing infective niches. In this way, the fungus may be acting similar to plant endophytes which inhabit a plethora of plant environments without causing visible disease and have far greater phenotypic plasticity than their true plant pathogen cousins which cause disease in a limited host repertoire [168]. Thus, acquisition of virulence traits during environmental switching from the soil to the mammalian host may be pathogenic fungi’s greatest virulence trait. Microevolutionary adaptation can occur after inoculation of environmental strains in the mammalian host by restricted mutations in genes known as virulence-adaptation genes such as the iron acquisition reductase, *FRE3* [169]. Evidence of microevolution within the environment and among strains is also suggested in genome sequencing and transcriptisome analyses [170]. Integrated environmental pressures by diverse environments thus contribute a synergistic impact on virulence in the fungus and may result in clonal outbreaks in humans as suggested recently for *Cryptococcal gattii* in the Pacific Northwest of North America [171].

## 6. Conclusions

Here, we have discussed our current understanding of mechanisms for immune surface masking of pathogens from both bacteria and fungus highlighting the role for extracellular capsules. Functional similarities exist between bacterial and fungal capsules, including sharing of some antigenic determinants of fungi with certain pneumococcal PSs. Capsules are widely acknowledged to be indispensable virulence factors and continue to provide insights into pathogenic mechanisms. Despite numerous studies, additional areas remain unexplored, especially regarding fungal capsules. Understanding characteristics that separate pathogenic and non-pathogenic microbial capsules is thus an important area of research for ascertaining the virulence potential of a wide array of bacterial and fungal capsular microbes.
